# On Population Growth Near Protected Areas

**DOI:** 10.1371/journal.pone.0004279

**Published:** 2009-01-26

**Authors:** Lucas N. Joppa, Scott R. Loarie, Stuart L. Pimm

**Affiliations:** 1 Nicholas School of the Environment, Duke University, Durham, North Carolina, United States of America; 2 Department of Global Ecology, Carnegie Institute, Stanford, California, United States of America; University of Exeter, Cornwall Campus, United Kingdom

## Abstract

**Background:**

Protected areas are the first, and often only, line of defense in efforts to conserve biodiversity. They might be detrimental or beneficial to rural communities depending on how they alter economic opportunities and access to natural resources. As such, protected areas may attract or repel human settlement. Disproportionate increases in population growth near protected area boundaries may threaten their ability to conserve biodiversity.

**Methodology/Principal Findings:**

Using decadal population datasets, we analyze population growth across 45 countries and 304 protected areas. We find no evidence for population growth near protected areas to be greater than growth of rural areas in the same country. Furthermore, we argue that what growth does occur near protected areas likely results from a general expansion of nearby population centers.

**Conclusions/Significance:**

Our results contradict those from a recent study by Wittemyer *et al.*, who claim overwhelming evidence for increased human population growth near protected areas. To understand the disagreement, we re-analyzed the protected areas in Wittemyer *et al.*'s paper. Their results are simply artifacts of mixing two incompatible datasets. Protected areas *may* experience unusual population pressures near their edges; indeed, individual case studies provide examples. There is no evidence, however, of a general pattern of disproportionate population growth near protected areas.

## Introduction

Protected areas are often the primary defense against species extinctions and habitat loss [1]. The global network of protected areas now covers more than 12% of the terrestrial earth surface [2]. With rapid population growth, human activity increasingly dominates landscapes surrounding this network [3]. Do protected areas influence human activity near their borders [4]? The answer is critical to assessing the effectiveness of protected areas towards conserving biodiversity as well as the contribution of biodiversity towards rural development.

Many argue that protected areas are detrimental to rural development by excluding people from traditional lands and further marginalizing them by denying access to natural resources [5], [6]. In that regard, there are concerns as to whether allocating resources away from rural economies, and towards biodiversity, is justified. In contrast, increasing numbers of rural people are moving to cities and towns in search of economic opportunities [7]. This argument would suggest that human activity and population growth near rural protected areas would be below the country average. Such a trend would benefit biodiversity. A recent study across African and Neotropical moist forests [8] found no evidence of increased deforestation near protected area boundaries, lending support for this argument.

Others suggest that protected areas provide benefits for rural communities [9]. Protected areas require infrastructure, such as roads leading to their entrance, and people to work in them. Natural areas also provide many ecosystem services [10], [11]. Whether used sustainably or not, protected areas are often the last remnants of natural resources available to rural communities. Theoretically, the combination of infrastructure, employment, and necessary goods and services could cause protected areas to serve as surrogate urban centers, attracting human settlement and population growth. Such a trend would be a testament to the value associated with ecosystem services, ecotourism, and natural resources for rural economies. This optimism comes at a cost. By encouraging population growth and accelerating the isolation of the protected area from natural landscapes, the net impact of protected areas on conserving biodiversity may be negligible.

Wittemyer *et al.* [12] claim to provide the first consistent evidence supporting this latter argument. The title of their paper, “Accelerated Human Population Growth at Protected Area Edges”, is a succinct summary of their results, claiming population growth rates near park boundaries are higher than national rural growth rates. The authors compared growth rates in a single 10 km buffer around each of 306 protected areas [2] in 45 African and South and Central American countries from geographically explicit data [13], [14] with a UN-supplied single estimate of the country's rural growth rate [15].

Using a more spatially explicit approach, we re-analyzed population growth around protected areas. There is no evidence to support disproportionate population growth near protected areas. There are systematic differences between the two independent datasets Wittemyer *et al*. used to generate the study's results and this discrepancy is sufficient to explain their results.

## Results

**Figure 1:**
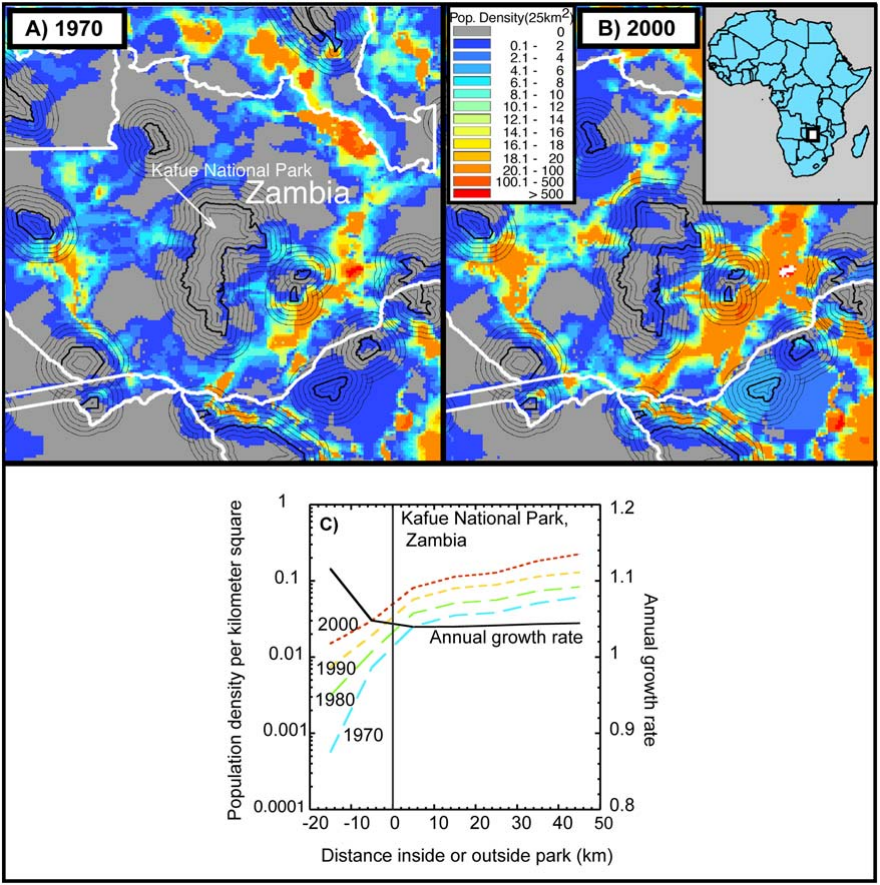
Changes in population density in and around Kafue National Park, Zambia, from 1970 to 2000. A, B) Population density around Kafue National Park (established 1971) in 1970 (A), and 2000 (B) expressed as people per 25 km^2^, the unit of analysis. Parks are outlined in heavy black, and lighter black lines radiating from them represent increasing 10 km intervals. From 1970 to 2000, growth in the buffer zone around Kafue increases because of increasing and multi-directional growth from pre-existing populations outside the 10 km buffer. C) Population densities at 10 km intervals inside and outside of Kafue National Park (left-hand y axis) and annual growth rates (right-hand y axis). We show densities for 1970, 1980, 1990, and 2000. There is no tendency for population densities to increase near the boundary.

Using methods we employ elsewhere [8], we create a series of 10-km wide buffers inside and outside of our sample of 304 protected areas (the same as those analyzed by Wittemyer *et al*.) and calculate the population densities within these annuli. This technique is necessary to avoid inherent problems with creating a single buffer, as doing so ignores events immediately outside the buffer. Figures 1a and 1b show these annuli around Kafue National Park (NP) in Zambia. We use Kafue NP as an example because Wittemyer *et al.* highlighted the area in their study and we have extensive experience working there.

Figure 1c plots the population densities against distance from 20 km inside to 50 km outside the boundary of Kafue NP. From these densities, we derive the corresponding annual growth rates directly. Plots similar to Fig. 1c for all 304 protected areas are available on request. As is clear in Figure 1c, annual growth rates remain virtually unchanged with increasing distance outside of Kafue National Park. High growth rates are sometimes found *inside* park boundaries (as in Kafue), but here data are very sparse and prone to measurement error. Our results for Kafue NP contradict those of Wittemyer *et al.*'s. We provide an explanation for this disagreement presently.

**Figure 2:**
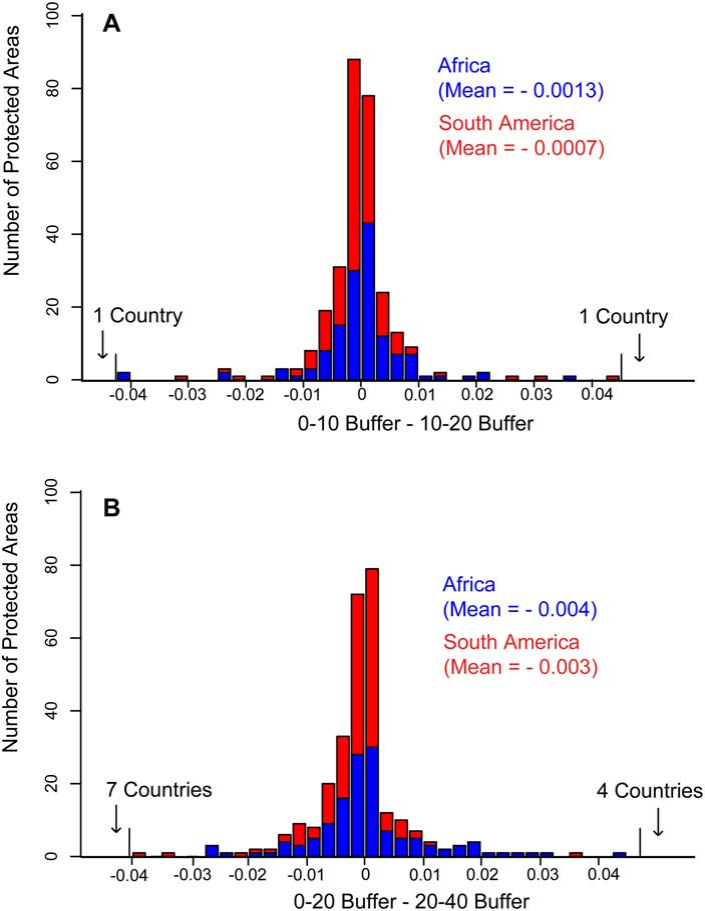
Differences in annual population growth at increasing distances from all 304 parks. A) Annual population growth in 10 km buffer zones minus the annual population growth in 20 km buffers on the x-axis, number of protected areas on the y-axis (304 parks). If 10 km buffers were experiencing accelerated growth, most values would be greater than zero. This is not the case. B) Same as for (A), but comparing 0–20 km buffer zones with 20–40 km buffer zones. Again, there is no evidence for disproportionate population growth near protected area boundaries.

It is difficult to convey results for all 304 protected areas in the same manner as Figure 1c. Nevertheless, Figures 2a and 2b provide an adequate summary for all the protected areas in our sample. If people were immigrating to protected area boundaries, population growth 0–10 km away from the boundary would be higher than growth 10–20 km away. (And, by extension 10–20 should be larger than 20–30, 0–20 larger than 20–40, and so on.)

If one assumes that protected areas draw people to them, then population growth of the 0–10 km buffer minus that in the 10–20 km buffer should be greater than zero. As found for Kafue, and shown for all 304 protected areas in Figures 2a and 2b, there is no tendency for growth rates to be higher adjacent to park boundaries (0–10 km) than further away (10–20 km) in either Africa (mean = −0.0013) or South America (mean = −0.0007). We repeat this analysis for the comparison of 0–20 km and 20–40 km buffers and find the same result (Figure 2b). Indeed, we have made many such comparisons and always find no differences. Such comparisons refute Wittemyer *et al.*, but match our previous results on land use changes near parks [8].

Although we find no evidence that population growth is disproportionate near protected area boundaries, populations are indeed growing. This is inevitable as human population continues to expand worldwide. Here it is the mechanism of the growth that matters, and again, Kafue NP provides an example (Figures 1a and 1b). If rural protected areas attract human settlement, one might expect isolated population centers to spring up, unassociated with preexisting population centers. This should be obvious through visual inspection.

Instead, what one sees around Kafue NP follows well-understood features of human demography. What growth does occur in buffers is often from the growth of existing population centers incidentally expanding towards protected areas. Figures 1a and 1b show this clearly for Kafue NP, where we map human density in the decade of Kafue NP's establishment (Figure 1a), and human density in the current decade (Figure 1b). When Kafue NP was established, there were few people living immediately outside the boundary, but several population centers existed at distances greater than 30 km to the northwest and east. Over time, these population centers grew multi-directionally. Kafue NP, which remained stationary on the landscape, was simply in the way of population expansion. Inspection of many other parks shows this to be a common trend.

### Wittemyer et al.'s results are artifacts of comparing incompatible data

To understand why Wittemyer *et al.* found spurious results, we repeated their analysis as best we could. Using their methodology, we closely matched their results and found 253 of 304 (83%) parks with higher growth in the buffers (obtained from one data set) compared to rural growth (from the other). This compares well with Wittemyer *et al.*'s 245 of 306 (80%) parks.

These results are artifacts of mixing two incompatible datasets. Wittemyer *et al.* calculated population growth rates near protected areas using geographically explicit data [13], [14], and compared these rates to a UN-supplied single estimate of the country's rural growth rate [15]. Unfortunately, the geographically explicit dataset provides consistently higher rural growth rates than does the UN-supplied one. We show this is true by deriving an alternative calculation of rural growth, one originally presented in Wittemyer *et al.*'s supplemental materials [12].

Using a global map of urban and rural extents, it is possible to mask out areas identified as “urban” in the geographically explicit data [16]. This is ideal, as doing so allows one to calculate rural growth rates from the same dataset used to calculate growth rates near protected areas. A comparison between UN-supplied rural growth rates and those independently derived from consistent datasets shows why Wittemyer *et al.*'s results could have been no other way.

As UN-supplied rural growth rates increase, so too did those from the country's synoptic data on rural growth [15] (Spearman's rank correlation: r = 0.501, n = 45, p<0.001). Wittemyer *et al.* found a similar correspondence (Spearman's rank correlation: r = 0.501, n = 45, p<0.001), and used this highly significant correlation to justify mixing the two datasets.

**Figure 3:**
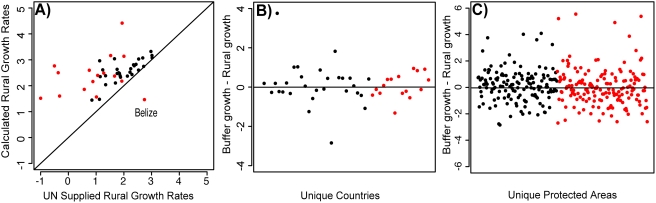
Replication and re-analysis of Wittemyer *et al.*'s methods and results. A) Evidence for the incompatibility of the two datasets used to calculate growth rates in Wittemyer *et al.*'s study. We plot rural growth rates from [15] on the x-axis, and rural growth rates derived from [13], [14], [16] on the y-axis. Red points represent South American countries, while black indicates African countries. Almost all (44 of 45) countries are above the 1∶1 line, indicating that the datasets used to calculate buffer growth rates provide consistently higher rural growth rates than does the UN dataset. The point below the 1∶1 line is Belize, one of only two countries in South and Central America where Wittemyer *et al.* failed to find a positive result. B) The difference between average growth rates in park buffer zones and rural growth rates for all 45 countries, when we calculate rural growth from the same dataset as buffer growth. C) The same as “B”, but this time displaying the results for 304 individual parks. Neither “B” nor “C” is statistically significant (p = 0.766, p = 0.774, respectively).

Unfortunately, what is needed here is not simply a strong correlation but a one-to-one correspondence. Figure 3 shows the strong correlation but also that the geographically explicit data are, with just one exception, higher than the UN-estimates. This relationship between the two datasets ensures Wittemyer *et al.*'s results.

We then repeated Wittemyer *et al.*'s analysis, calculating both rural and buffer growth rates from [13], [14], [16]. Using these derived rural growth rates, we calculated dramatic differences from Wittemyer *et al.*'s main results. Using incompatible datasets, they found buffer growth to be higher than rural growth for 245 of 306 (80%) parks and 38 of 45 (84%) countries. In Figures 3b and 3c, we show that by using a single dataset those results reduce to 155 of 304 (51%) parks and 24 of 45 countries (53%). There are no more parks with higher growth rates near them than parks with lower growth rates (p = 0.774, p = 0.766, respectively; binomial test).

## Discussion

Do protected areas, and their perceived benefits, attract people to them? The question is of utmost importance. Conservation efforts can draw both positive and negative actors to the scene. In a manner similar to locating the last remaining population of an endangered species, the creation and funding of a protected area could potentially cause more harm than ignoring the area altogether [4]. We find no evidence that human population growth near protected area boundaries is higher than in rural areas and show that Wittemyer *et al.*'s counter-result is methodologically flawed.

The geographically explicit data are not raw population counts, but predictions from a complex model that, in perhaps indirect and complex ways may include the proximity of a park boundary as a factor. Original population data comes from the level of census unit. The average resolution for all African countries (excluding South Africa) is 82 km^2^/census unit. Some countries, such as Chad and Angola, have much lower resolutions (303 and 263 km^2^/census unit, respectively). While individual maps appear highly resolved, much of this cancels out when one divides the two datasets to calculate growth rates. Maps of growth collapse to the coarse level of census unit or lower, calling into question the suitability of these modeled population datasets for fine spatial-scale analyses. As these numbers show, the data are likely too sparse to draw fine-scale conclusions about population growth. In contrast, our analyses of land-use changes are consistently of 1 km^2^ resolution [8].

More generally, the scarcity of suitable datasets poses a challenge for conservation [17] and we note the need for more high-resolution biological and social data.

When assessed using much finer-scale metrics such as land-cover, we find that protected areas perform admirably [8]. However, efforts to keep protected areas protected must increase as the global network becomes increasingly isolated [3] and ever more in contact with growing human populations. As both the protected area network and human population grow, collisions between these areas and people struggling to find land on which to survive will continue. Kafue NP (Figures 1a and 1b) provides an example of this. Connecting existing protected areas through corridors [18], creating future protected areas in places they can be most effective [1], and effectively managing all protected lands will be essential to ensure the future of biodiversity.

## Methods

All datasets are global in scale, in raster (grid) format, and projected into Albers Equal Area projection at a resolution of 5km grid square (25 km^2^). We used ArcGIS 9.1 to harmonize projections, cell size, and extent across datasets. We carried out all further analyses in R 2.6.

We obtained information on park location from the 2007 World Database on Protected Areas [2]. The 304 protected areas in our analysis are a sample of the 306 protected areas included in the analysis by Wittemyer *et al.* A full description of the criteria used to choose the sample of protected areas can be found in their supplemental materials [12], but in brief Wittemyer *et al.* only chose areas greater than 10 km^2^, established before 1995, not on oceanic islands, and IUCN category I or II (non-consumptive use categories) or World Heritage Sites. They also excluded protected areas with no people in the 10 km buffer zone at the time of protected areas establishment, or with urban settlements greater than 1000 people.

We calculated human population growth rates using decadal modeled population datasets for Africa [13] and South and Central America [14]. To replicate Wittemyer *et al.*'s results, we obtained country-specific rural growth rates from [15]. To calculate rural growth rates from the decadal population datasets, we masked out all areas identified as “urban” by [16]. Wittemyer *et al.* provide further details of the analysis in their supplemental materials [12].

We then created 10 km wide annuli in and around each protected area, from 20 km inside the protected area to 50 km outside. Using the decadal datasets, we were able to calculate growth rates at ten-year intervals for each of the annuli. We obtained the annual growth rate by dividing the total growth rate by the number of years the analysis encompassed. In order to summarize these results, for each protected area we then divided the growth rate in the 0–10 km buffer by the growth rate in the 10–20 km buffer. Values greater than one indicate higher population growth near protected areas than away. When repeating Wittemyer *et al.*'s analysis, we followed their methodology of calculating population growth inside the 0–10 km buffer using the decadal datasets [13], [14] and subtracting from that the UN-supplied rural growth rate of the country [15]. Positive values indicate protected areas with higher human population growth than rural areas of the same country.
